# Kidney Tissue Targeted Metabolic Profiling of Unilateral Ureteral Obstruction Rats by NMR

**DOI:** 10.3389/fphar.2016.00307

**Published:** 2016-09-15

**Authors:** Zhenyu Li, Aiping Li, Jining Gao, Hong Li, Xuemei Qin

**Affiliations:** ^1^Modern Research Center for Traditional Chinese Medicine, Shanxi UniversityTaiyuan, China; ^2^Shanxi Hospital of Integrated Traditional and Western MedicineTaiyuan, China

**Keywords:** ^1^H NMR, metabolomics, renal interstitial fibrosis, unilateral ureteral obstruction, kidney

## Abstract

Renal interstitial fibrosis is a common pathological process in the progression of kidney disease. A nuclear magnetic resonance (NMR) based metabolomic approach was used to analyze the kidney tissues of rats with renal interstitial fibrosis (RIF), induced by unilateral ureteral obstruction (UUO). The combination of a variety of statistical methods were used to screen out 14 significantly changed potential metabolites, which are related with multiple biochemical processes including amino acid metabolism, adenine metabolism, energy metabolism, osmolyte change and induced oxidative stress. The exploration of the contralateral kidneys enhanced the understanding of the disease, which was also supported by serum biochemistry and kidney histopathology results. In addition, the pathological parameters (clinical chemistry, histological and immunohistochemistry results) were correlated with the significantly changed differential metabolites related with RIF. This study showed that targeted tissue metabolomic analysis can be used as a useful tool to understand the mechanism of the disease and provide a novel insight in the pathogenesis of RIF.

## Introduction

Renal interstitial fibrosis (RIF) is a common histopathological change and the major determinant in the progressive chronic kidney diseases that lead to end-stage renal failure. It ultimately requires dialysis or kidney transplantation if not treated with effective therapy interventions and poses a serious threat to the quality of human life and health (Zhang et al., 2012).

Unilateral ureteral obstruction (UUO) is a well-documented hydronephrosis model simulating RIF. RIF is characterized by a rapid consequence of events: first, UUO leads to reduced renal blood flow and glomerular filtration rate; second, hydronephrosis, interstitial inflammatory infiltration, and tubular cell death happen; UUO also promotes the renin-angiotensin system and then it triggers epithelial-mesenchymal transition (EMT); cytokines and growth factors are then produced and extracellular matrix is accumulated; finally, the continuation of hydronephrosis results in tubulo-interstitial fibrosis and the progressive loss of renal function (Lee et al., 2014).

Over the last few years, numerous studies have been conducted to identify the molecular mechanism of anti-fibrotic effect, which can attenuate or reverse RIF by interfering with several aspects of the pathogenic processes. These include regulating profibrotic markers, such as α-SMA (Yuan et al., 2011), TGF-β1, and HGF (Zuo et al., 2009), inflammatory markers, such as ED-1 and MCP-1(Yuan et al., 2011), nuclear translocation of Nrf2 (Chung et al., 2014), blocking of tubular epithelial-myofibroblast transition (TEMT; Xie et al., 2008), enhancing NO production via eNOS activation and scavenging ROS (Meng et al., 2007), degrading extracellular matrix and collagen deposition, and so on (Xie et al., 2009). These considerable efforts have laid the foundation for exploring this disease and designing the related therapy.

Metabolomics can provide crucial global insight into stimuli-induced metabolism shift in different physiological or pathological states before histopathological changes are detected. Nuclear magnetic resonance (NMR) allows for rapid, nondestructive, nonselective and highly reproducible data collection for qualitative and quantitative metabolite analysis. ^1^H NMR-based metabolomics have been used in many renal related studies, such as identification of the pathogenesis of diabetic nephropathy (Wei et al., 2015), evaluation of renal transplant dysfunction (Serkova et al., 2005), prediction of renal function after obstruction relief (Dong et al., 2013) and detection of drug induced nephrotoxicity (Sieber et al., 2009). Metabolomic analysis of RIF using UUO model has extracted significant biological information and assessed the endogenous metabolite changes in serum, urine, and kidney samples, which has deepened the understanding of the pathogenesis of RIF and offered invaluable information for diagnosis and therapy treatment of RIF (Maclellan et al., 2011; Zhang et al., 2012; Fang et al., 2016; Xiang et al., 2016; Zhao et al., 2016).

In the present study, ^1^H NMR based-metabolomic approach was applied on both the left and right kidney tissue of UUO rats to characterize the alteration of renal endogenous metabolites. Potential biomarkers related with RIF were identified by multivariate analysis, and the corresponding metabolic networks were built in Metscape to elucidate the progression of RIF. The metabolic change between the bilateral kidneys was compared to further elucidate the molecular mechanism of RIF progress. In addition, the relationship between the traditional pathological indexes (histological change, clinical chemistry, and protein expression) and the perturbed endogenous renal metabolites was also studied.

## Materials and methods

### Chemicals

Methanol, Na_2_HPO_4_ and KH_2_PO_4_ were purchased as analytical grade from Beijing Chemical Works (Beijing, China). Sodium 3-trimethlysilyl [2,2,3,3-d_4_] propionate (TSP) was from Cambridge Isotope Laboratories Inc. (Andover, MA, USA). D_2_O was obtained from Norell (Landisville, Pennsylvania, USA). Phosphate buffer, prepared by dissolving KH_2_PO_4_ and Na_2_HPO_4_ in water (0.2 M, pH 7.4) containing 0.01% TSP and 10% D_2_O, was used to reconstructed tissue extract for NMR analysis. Bull Serum Albumin (BSA), primary antibodies, including rabbit antibodies to HGF (Santa Cruz Biotechnology, Santa Cruz, CA, USA), rabbit antibodies to TGF-β1 (Santa Cruz Biotechnology), secondary antibody: polymerized HRP, 3,3′-diaminobenzidine (DAB), Eukitt mounting medium (Sigma-Aldrich), Tris Buffered Saline (TBS), and phosphate buffer (PBS) were bought from BosterBio Co., Ltd (Wuhan, China).

### Ethics statement

The study was approved by the Experimental Animal Ethical Committee of Modern Research Center for Traditional Chinese Medicine, Shanxi University. All experiments were performed in strict compliance with the recommendations in the Guide for the Care and Use of Laboratory Animals of the National Institutes of Health (NIH) considering animal experiments, as well as the internationally accepted ethical principles for laboratory animal use and care.

### Animal surgery and sample collection

Male Sprague Dawley rats, weighing 180–200 g, were commercially obtained from Beijing Vital River Laboratories [Co. SCXK (Jing) 2015-0014]. The animals were kept under a 12 h light/dark cycle, temperature (20–25°C) and constant humidity (50 ± 10%) in SPF (Specific Pathogen Free) grade laboratory conditions and were allowed to have free access to food and water.

After 1 week's adaptation, the rats were randomly divided into 2 groups: UUO group (model group, *n* = 11) and sham-operated (SO) group (control group, *n* = 9). The procedures on the UUO model rats were carried out according to the operating procedure described previously (Xiang et al., 2016). In brief, under 20% ethyl carbamate induced anesthesia (1.5 g/kg body weight), the left ureter was isolated, and completely ligated with 3-0 silk suture at two points and cut between the ligatures to prevent retrograde urinary tract infection. The SO rats underwent an identical surgical intervention except for ureter ligation.

**Figure 1:**
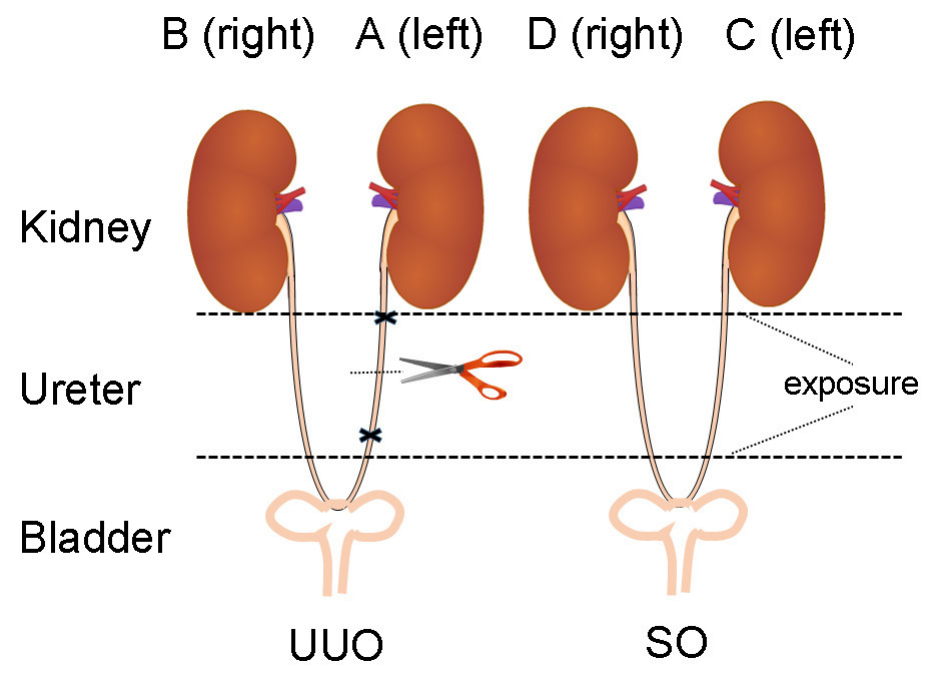
**The diagram of animal surgery for UUO model**. The left, UUO: the ureter is ligated and cut. The right, Sham controls (SO): the ureter is exposed, but not ligated (We express thanks to the authors for their idea/contribution on the diagram of animal surgery for UUO model, Ucero et al., 2014).

Blood samples were collected into EP tubes and centrifuged (3500 rpm, 15 min) to obtain serum. A 250 μL aliquot of serum was used for clinical biochemistry analysis. Kidney tissue was removed and weighed. One half of the left (A) and the right (B) kidney of UUO and SO (C and D) groups (Figure 1) were fixed in neutral buffered formalin and embedded into paraffin. The remaining kidney tissue was rinsed and snap-frozen in liquid nitrogen and stored at −80°C.

### Clinical chemistry

The clinical chemistry analysis of serum was carried out for the measurement of biochemical parameters including serum creatinine (Scr), blood urea nitrogen (BUN), and albumin (ALB) by using Automatic Analyzer (PRIME60i, Thermo Fisher, USA). A value of *p* < 0.05 using *t*-test was considered to be significant.

### Histopathological assessments and immunohistochemistry

Histologic analyses were performed using paraffin-embedded tissues. To evaluate the severity of tubular injury and tubulointerstitial fibrosis, kidney sections were processed and stained with hematoxylin and eosin (H&E) and Masson trichrome, respectively. Renal tubular injury was assessed and given a score defined as tubular injury score (TIS) from 0 to 3 (0 = normal, 1 = slight damage, 2 = moderate damage, 3 = severe damage) as previously described (Wang et al., 2016). The extent of interstitial collagen deposition (ICD) was evaluated according to literature (Xie et al., 2008).

Sections were deparaffinized, rehydrated, and washed in PBS. After endogenous peroxidase was blocked by incubation with 3% H_2_O_2_ for 10–15 min, heat-induced antigen retrieval was achieved by 4-min autoclaving in 10 mM citrate buffer (pH 6.0). For detection of TGF β1 and HGF, nonspecific binding sites were blocked with 5% BSA in PBS for 30 min, and the sections were incubated overnight in a humidified chamber at 4°C with primary antibody. After rinsing in PBS, the sections were incubated with the secondary antibody for 30 min at room temperature and subsequently washed with PBS. For coloration, the sections were incubated with a mixture of 0.05% 3,3′-diaminobenzidine (containing 0.03% H_2_O_2_) at room temperature until a brown color was visible, and then washed with PBS (pH 7.4), counter stained with hematoxylin, dehydrated, and mounted in Eukitt mounting medium (Sigma-Aldrich). Then they were observed under light microscopy (CX31-12C04, Olympus, Japan).

### Samples preparation for NMR analysis

Kidney tissues were weighted and extracted in a ratio of methanol and water (2:1) by use of an Ultrasonic cell crusher (Ningbo Scientz biotechnology Co., LTD, China) according to the literature (Ghosh et al., 2013) with minor adjustment. The supernatants were freeze-dried and dissolved in 700 μL phosphate buffer (0.1 M, KH_2_PO_4_/Na_2_HPO_4_, pH 7.4) containing 0.01% TSP and 10% D_2_O. After centrifugation (13,000 rpm, 4°C, 10 min), 600 μL of supernatant was transferred into a 5 mm NMR tube for analysis.

### NMR measurement

^1^H NMR spectra was acquired on a Bruker 600-MHz AVANCE III NMR spectrometer (Bruker BioSpin, Germany) at 298 K, using the noesygppr1d pulse sequence for water suppression. 64 scans were collected into 65,536 data points over a spectral width of 12,345.7 Hz, with a relaxation delay of 1.0 s, and an acquisition time of 2.65 s.

### Data reduction and multivariate pattern recognition analysis

All spectra were carefully phased and baseline corrected and referenced to TSP at δ 0.00 using MestReNova (version 8.0.1, Mestrelab Research, Santiago de Compostella, Spain). Region distorted by residual water (δ 4.68–5.23) was excluded in the subsequent analysis. Then, all spectra were segmented at 0.01 ppm (buckets) across δ 0.78–9.66 and normalized to tissue weights. The normalized integral values were then subjected to multivariate data analysis using Simca-P 13.0 software (Umetrics, Sweden). Principal component analysis (PCA) was performed on the mean centered data to generate an overview for group clustering and to search for possible outliers. Following validating the quality of the classification PLS-DA model with *R*^2^ and *Q*^2^, metabolomic alteration was revealed by the orthogonal projection to latent structure with discriminant analysis (OPLS-DA; Bylesjö et al., 2006) with cross-validation. The validity of the model is evaluated by the CV-ANOVA method at the level of *p* < 0.05 (Hao et al., 2016). The corresponding S-plots, where each point represents a single NMR spectral region segment, were used to identify which spectral variables contributed to the separation of the samples on the scores plot (Ji et al., 2016). Variable importance in the projection (VIP) values and coefficients were also applied to screen the important variables. ANOVA at confidence intervals of 95% using SPSS 16.0 software was used to evaluate the statistical significance based on the normalized integral values selected from the least overlapping NMR signals.

### Computational data analysis

Metscape, the metabolic network analysis and visualization tool (http://metscape.ncibi.org./, Gao et al., 2010), was used to generate the compound network associated with each of the differentiating metabolites. Metscape is a plugin for Cytoscape (http://www.cytoscape.org/). This tool uses Kyoto Encyclopedia of Genes and Genomes (KEGG) ID as the primary compound identifier and contains nearly 2700 compounds, 870 enzymes, 1400 genes, and 3000 metabolic reactions involved in over 70 human-specific metabolic pathways defined in the Edinburgh Human Metabolic Network (EHMN; Ma et al., 2007).

Compound concentration data (including compound IDs, significance values, and fold change values) were loaded from file in CSV format. Then “compound network,” one of the canonical network type, was selected to query the internal relational database (KEGG and EHMN) and create the correlation networks. The networks included all uploaded differential compounds and any neighboring compounds when a species [Rattus norvegicus (Norway rat)] was chosen, which contributed to identifying enriched pathways from expression profiling data, and visualizing changes in metabolite data.

## Results

### Kidney morphological appearance and size

Both kidneys in SO rats (C and D) showed normal appearance and size with tissue ruddy and no swelling (Ucero et al., 2014). While for UUO group, the size of left kidney (A) increased significantly, and kidney hydrops appeared, renal parenchymal degenerated, renal pelvis dilated, and petechial hemorrhages appeared sporadically. Compared with SO rats, the size of right kidney (B) in UUO rats also increased (B, 1.67 ± 0.23 g; D, 1.18 ± 0.09 g).

### Clinical chemistry

Standard clinical chemistry measurements of renal function such as Scr, BUN, and ALB were taken to provide an indication of “renal status” of the animals. As shown in Table S1, UUO rats showed significant elevations of serum Scr and BUN accompanied with level decrease of ALB. And the level change of serum Scr and BUN were bigger than that of ALB.

### Histology and immunohistochemistry

**Figure 2:**
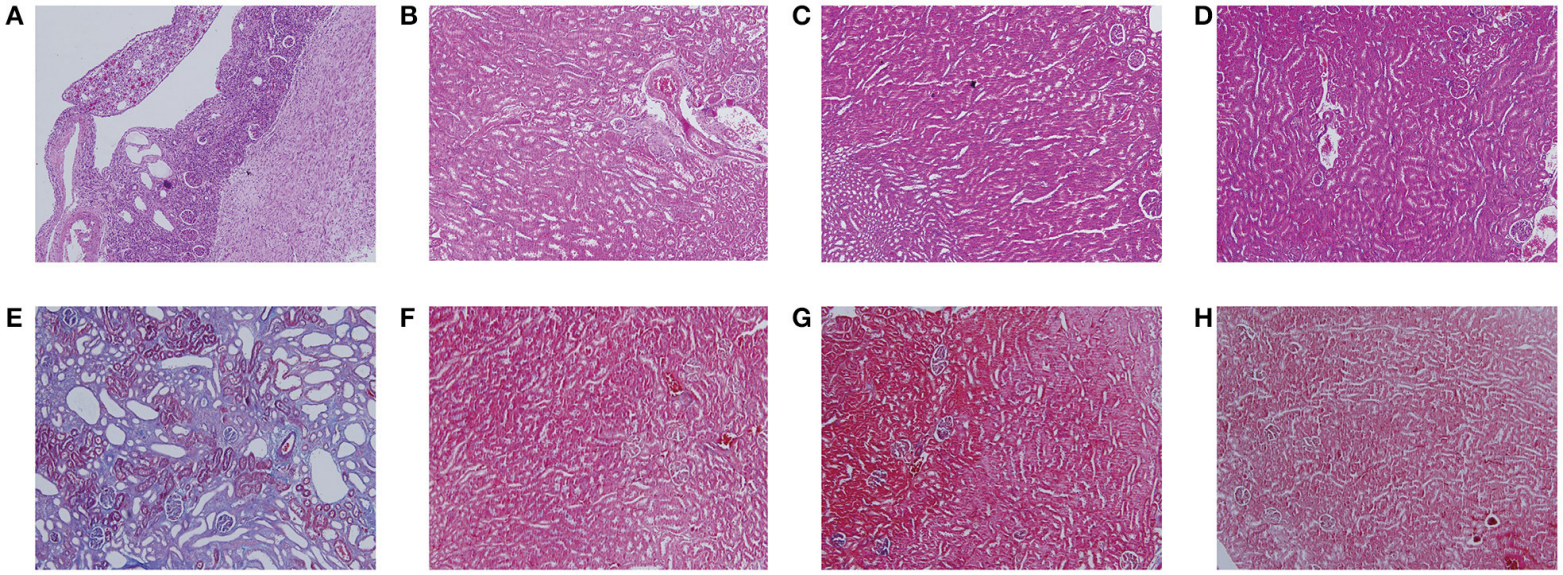
**Histological features of mouse kidney after ureteral obstruction**. Hematoxylin and eosin (H&E) **(A–D)** and Masson's trichrome (Masson) staining **(E–H)** illustrate the main tubulointerstitial histological changes (renal tubular injury and collagen deposition) in the obstructed kidney. **(A,E)** the left kidney of UUO rat, **(B,F)** the right kidney of UUO rat, **(C,G)** the left kidney of SO rat, and **(D,H)** the right kidney of SO rat.

Figure 2 showed the representative images of the histological examination of H&E-stained and Masson-stained sections of both left and right kidneys of UUO and SO rats. The left kidney of UUO rats showed a high TIS characterized by tubular dilatation and atrophy, interstitial inflammation, and a marked interstitial fibrosis by H&E staining (Table S2). For the UUO rats, the histologic damage with ICD determined by Masson staining (Table S2) was much server in the left kidney than that of the right kidney, whereas both SO kidneys (left and right) exhibited relative normal histopathology.

TGF-β1 is known to be the most important cytokine involved in epithelial-to-mesenchymal transition (EMT) and fibrosis in a variety of animal models. A drastic increase of TGF-β1 is a key feature of the kidney in the UUO rats (Zuo et al., 2009). As shown in Figures S1A–D, TGF-β1 was mainly expressed in tubular and interstitial cells in the left kidney of UUO rats, indicated by immune histochemical staining. HGF has been identified as an antifibrotic molecule and an antagonist of TGF-β1, and possessing remarkable ability to promote tissue repair and regeneration after various injuries (Liu, 2004; Liu and Yang, 2006). The levels of HGF protein expression in the left kidney of UUO rats decreased dramatically (Figures S1E–H). However, there were no significant differences in the expression levels of TGF-β1 and HGF between the left and right kidney of SO rats (C and D). In addition, the expression level of TGF-β1 was also elevated in the right kidney of UUO rats as compared with SO group, while the HGF protein expression level was similar to that of SO group.

### UUO induced metabolomic changes in kidney tissue

**Figure 3:**
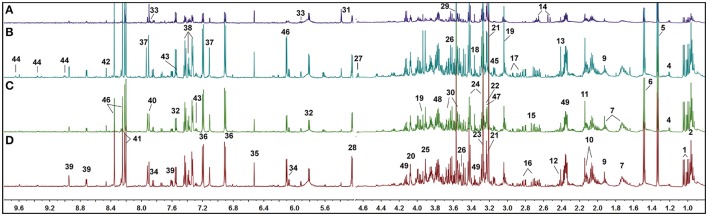
**Representative ^1^H NMR spectra of rat kidney extracts showing the left kidney of UUO rat (A), the right kidney of UUO rat (B), the left kidney of SO rat (C), and the right kidney of SO rat (D)**.

Typical ^1^H NMR spectra of kidney tissue extracts were shown in Figure 3. Resonance assignments (Table S3) were performed on the basis of the chemical shifts of standard compounds from the Chenomx NMR suite (evaluation version, Chenomx Inc., Canada), the Human Metabolome Database (HMDB, http://www.hmdb.ca/; Wishart et al., 2009), and Biological Magnetic Resonance Data Bank (BMRB, http://www.bmrb.wisc.edu/; Markley et al., 2007), as well as the literature data (Peiqiu et al., 2009; Niu et al., 2016). In addition, 2D HSQC (Figure S2) and COSY (Figure S3) were also used to confirm the NMR assignment. The cross-peaks in HSQC were manually picked, and 370 peaks in [^1^H, ^13^C] format were input in COLMAR ^13^C−^1^H Query server (http://spin.ccic.ohio-state.edu/index.php/hsqc/; Bingol et al., 2014). The cutoff value for ^1^H and ^13^C were set as 0.06 and 0.6 ppm, respectively. The results were manually checked by interactive user interface using the “Show Me” button, as well as the parameter of Matching ratio and Uniqueness. The NMR spectra of kidney were dominated by peaks from amino acids, organic acids (acetate, citrate, succinate, and lactate, etc.), choline-containing metabolites, nucleotide metabolites such as uracil, cytidine and xanthine, and other metabolites such as nicotinamide and betaine.

Visual inspection of ^1^H NMR spectra of rat kidney extracts indicated that UUO induced obvious metabolic variations, such as the elevation of lactate and allantoin, as well as the decrease of TMAO, adenosine, tyrosine, and valine and leucine. In order to disclose the minor differences between the UUO and SO groups, and to identify the potential marker metabolites associated with RIF, the ^1^H NMR spectra of kidney were segmented and subjected to multivariate statistical analysis.

**Figure 4:**
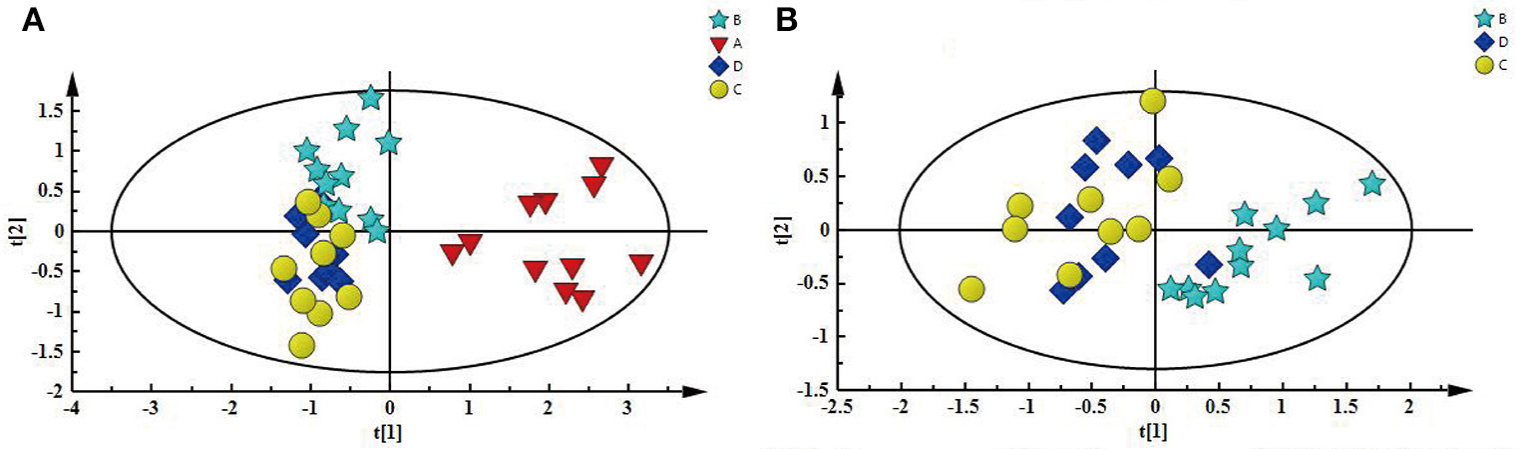
**(A)** PCA score plot depicting a separation of A and the three other groups, **(B)** PCA score plot showing difference between B and SO (C and D).

First, PCA model, an unsupervised multivariate data analysis method, was constructed to obtain an overview of the differences/similarities among the different groups of kidney extracts, in which PC1 accounted for 56.8% of the total variance, and PC2 accounted for 14.2% of the total variance. As shown in Figure 4A, four different groups of kidney extracts can be separated into two clusters, the left kidney of UUO rats (A) was separated from the other three groups of kidney along PC1, while the right kidney of UUO rats (B) and both the left and right kidney of the SO rats (C and D) can be further separated by PC2, indicating that the metabolic profile of both left and right kidneys of UUO rats have been changed after the ligation of left ureter. The clustering pattern is in agreement with the results of histopathological assessments and TGF-β1 protein expression.

To explore the potential pathogenesis of RIF, the left kidneys of UUO (A) and SO (C) were compared by PCA (PC1: 0.674%; PC2: 0.123%) and a clear separation was observed (Figure S4A). Then partial least squares discriminant analysis (PLS-DA) model was further constructed and validated using the response of the permutation test through 200 permutations. And the model, in which *Q*^2^ line intercepts the Y axis at a negative value or the *Q*^2^-values obtained from the permutation model to the left are lower than the original points to the right, is deemed to be of great predictive ability and reliability (Li et al., 2014; Figure S4B). Potential biomarkers associated with RIF induced by UUO were further identified by OPLS-DA. It is also a supervised multivariable statistic method, and can separate predictive from non-predictive (orthogonal) variation to achieve a maximum discrimination between classes (Figure S5A). The parameters (*R*^2^ = 0.952, *Q*^2^ = 0.911, *p*-value of 1.00874e-007 certified by CV-ANOVA method) indicated the validity of the OPLS-DA model. The corresponding S-plot (Figure S5B), in combination with the VIP and coefficients values, was used to find metabolites contributing to the separation. In addition, *t*-test was also performed to test the statistical significance of the altered metabolites. Compared with the SO rats, the left kidney of UUO rats exhibited level increase of lactate, methionine, aspartate, allantoin, uracil, 3-HB, and level decrease of TMAO, leucine, valine, lysine, adenosine, hypoxanthine, tyrosine, and phenylalanine (Table S4).

**Figure 5:**
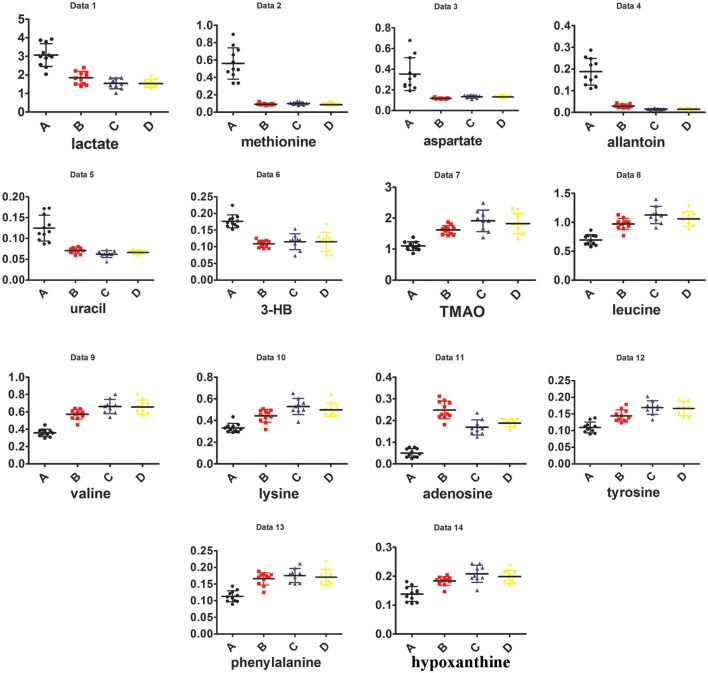
**The dot plots showing the levels of differential metabolites among A, B, C, and D**.

In addition, as shown in Figure 4B, the right kidney of UUO rats also differed from those of SO rats. The partially overlapped left and right kidney of the SO rats, indicated that minor difference existed between them. However, the permutation test (Figure S6) showed the corresponding PLS-DA model (left vs. right kidney of SO rats) was invalid, suggesting that operation induced metabolic difference between the left and right kidney can be neglected. Thus, the metabolic change of the right kidney in the UUO group was also induced by the ligation of left ureter. The significantly changed metabolites in the left kidney of UUO rats were relatively quantified in the four groups of kidney (Figure 5). Compared to the SO rats, metabolic changes in the right kidney of UUO rats were mainly involved in the decrease of TMAO, leucine, valine, lysine, hypoxanthine, and tyrosine, as well as the increase of lactate, uracil and allantoin. The changes of these metabolites showed the same trend as those in the left kidney, suggesting that moderate RIF also occurred in the right kidney of UUO rats. The average degree of fibrosis of the right kidney in the UUO rats was calculated as 25.5% (Table 1) according to the Equation (1) below, in which XAi and XBi denote relative contents of the metabolites in the left and the right kidney of UUO rats respectively, XCi denotes relative contents of the metabolites in the left kidney of SO rats, and α stands for change rate of biomarkers.

$$\begin{array}{l} \%\text{ average degress of fibrosis}=\frac{1}{n}\sum_{i\;=\;1}^{n\;=\;11}\left|\alpha \right|\times \text{100}\%\\ \;=\frac{1}{n}\sum_{i\;=\;1}^{n\;=\;11}\left|\frac{XBi-XCi}{XAi-XCi}\right|\times \text{100}\% \end{array}$$

**Table 1:** **Comparison of differential metabolites among different groups and the degree of changed metabolites**.

**No**.	**Name**	**C**	**A**	**B**	**A vs. C**	**B vs. C**	**α**	**% ADF**
1	Lactate	1.540	3.063	1.842	2.590E-06	0.047	0.198	
2	Methionine	0.099	0.561	0.092	6.711E-06	0.316	−0.015	
3	Aspartate	0.134	0.354	0.119	0.001	0.015	−0.069	
4	Allantoin	0.015	0.187	0.029	2.960E-06	0.000	0.082	
5	Uracil	0.062	0.125	0.071	3.879E-05	0.028	0.133	
6	3-hydroxybutanoate	0.115	0.177	0.109	1.364E-05	0.473	−0.104	
7	Phenylalanine	0.176	0.113	0.166	1.538E-06	0.791	0.155	
8	TMAO	1.914	1.104	1.618	5.690E-05	0.036	0.365	
9	Leucine	1.126	0.697	0.972	4.561E-06	0.018	0.360	
10	Valine	0.661	0.358	0.572	5.454E-07	0.016	0.294	
11	Lysine	0.530	0.332	0.443	1.383E-05	0.013	0.437	
12	Tyrosine	0.169	0.109	0.144	3.710E-06	0.012	0.415	
13	Hypoxanthine	0.208	0.138	0.184	4.507E-05	0.046	0.352	
14	Adenosine	0.169	0.051	0.249	9.880E-07	0.000	−0.680	
								0.255

### Correlation analysis between endogenous metabolites and renal function

The biplot (Figure S7) obtained from PLS can be used to describe the relationship among all the variables, including the potential biomarkers of RIF (X variables), the grouping or cluster (observation), and the data of clinical biochemical and histopathology (Y variables). The clusters of variables around the samples showed a positive correlation with them and the intensity of correlation was expressed with correlation coefficient (Table 2).

**Table 2:** **Correlation between the differential metabolites and the data of clinical biochemical and histopathology**.

	**Scr**	**BUN**	**ALB**	**TIS**	**ICD**
Lactate	0.667^**^	0.657^**^	−0.325	0.855^***^	0.851^***^
Methionine	0.842^***^	0.513^*^	−0.359	0.670^**^	0.873^***^
Aspartate	0.855^***^	0.602^**^	−0.403	0.750^***^	0.696^***^
Allantoin	0.784^***^	0.460^*^	−0.388	0.774^***^	0.892^***^
Uracil	0.811^***^	0.546^*^	−0.221	0.826^***^	0.809^***^
3-HB	0.824^***^	0.620^**^	−0.341	0.779^***^	0.832^***^
TMAO	−0.775^***^	−0.672^**^	0.358	−0.709^***^	−0.859^***^
Leucine	−0.760^***^	−0.815^***^	0.553^*^	−0.749^***^	−0.884^***^
Valine	−0.800^***^	−0.765^***^	0.564^*^	−0.750^***^	−0.928^***^
Lysine	−0.649 ^**^	−0.644^**^	0.463^*^	−0.785^***^	−0.869^***^
Adenosine	−0.769^***^	−0.682^**^	0.420	−0.651^**^	−0.903^***^
Tyrosine	−0.744^***^	−0.771^***^	0.533^*^	−0.739^***^	−0.864^***^
Phenylalanine	0.703 ^**^	0.480^*^	−0.338	0.798^***^	0.886^***^
Hypoxanthine	−0.794^***^	−0.610^**^	0.378	−0.632^**^	−0.799^***^

* *p < 0.05*,

** *p < 0.01*,

*** *p < 0.001*.

As shown in Figure S7, the clustering pattern of the left and right kidney of the UUO and SO rats was the same as that in Figure 4A. Higher serum Scr and BUN levels, TIS from the H&E-stained and ICD (%) from Masson-stained were positively correlated with the higher concentration of lactate, methionine, aspartate, allantion, uracil, and 3-HB in the left kidney of UUO rats. The left and right kidney in SO group contained more TMAO, leucine, valine, lysine, hypoxanthine, and tyrosine, all of which have a positive correlation with the serum ALB. It is interesting that the altered endogenous metabolites were positively correlated with the traditional indicators from clinical biochemical and histopathology, suggesting that metabolic profiling combined with multivariate statistics, which served as a global and sensitive method, has the potential to investigate the pathogenesis of diseases based on the disturbed endogenous metabolites.

## Discussion

Fibrosis is a dynamic system that involves consecutive events including apoptosis, necrosis, and proliferation (Chevalier et al., 2009). The tubulointerstitium accounts for 90% of the volume of the kidney (Hewitson, 2009), and thus detecting the metabolic changes of RIF could help to enhance the understanding of pathogenesis of renal fibrosis. The altered metabolic profiles in kidney, the target tissue that is of direct disease relevance, can provide important biological information regarding the ongoing status of kidney function. And it may facilitate understanding the pathological mechanism of early renal injury and provide the theoretical basis for the prevention or treatment of RIF.

**Figure 6:**
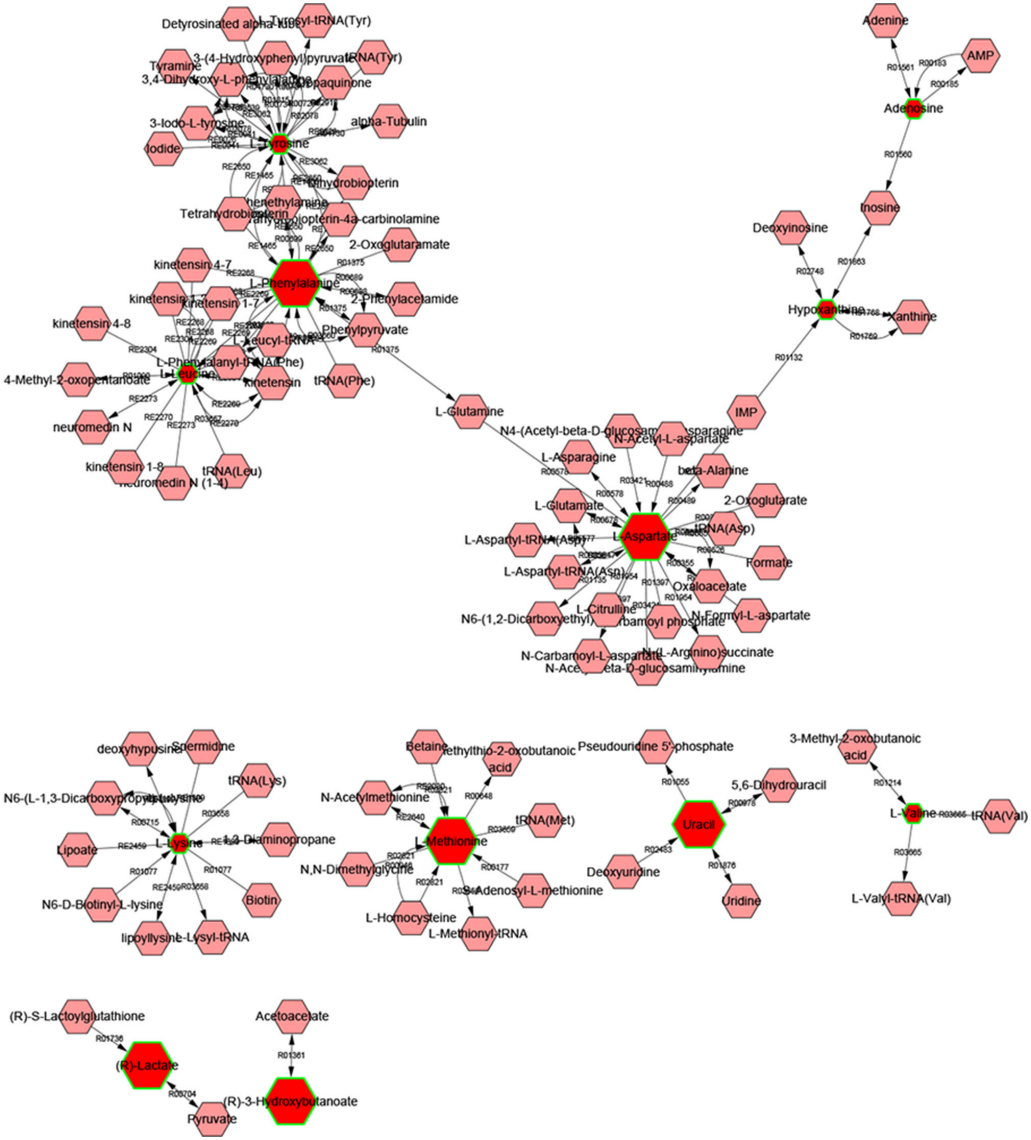
**The network of potential biomarkers associated with RIF by metscape analysis**.

Metscape is a tool for interactive exploration and visualization of experimental metabolomics and gene expression data in the context of human metabolic networks. It allows users to build and analyze networks of genes or compounds, identify enriched pathways from expression profiling data, and visualize changes in metabolite data. To gain additional insight about the relationship between metabolites, 14 statistically significant differential biomarkers were mapped to KEGG IDs and built compound network (Figure 6) consisting the nodes (metabolites) and edges that represent biochemical reactions. The network reflected complex pathology and provide evidence for the involvement of amino acid metabolism (1. phenylalanine, tyrosine, and tryptophan metabolism, 2. valine, leucine, and isoleucine biosynthesis, 3. tyrosine metabolism, 4. Methionine, and cysteine metabolism, 5. glutamate, and aspartate metabolism), purine metabolism, pyrimidine metabolism, glycolysis, and gluconeogenesis.

### Alteration in amino acid pathway induced by UUO

Amino acids are basic units for protein synthesis in organism. Increasing evidence showed that renal injury is highly associated with abnormality in protein expression and amino acids re-absorption (Remuzzi, 1995).

Phenylalanine is an essential amino acid, and its hydroxylation by phenylalanine hydroxylase to tyrosine is the major metabolic pathway for phenylalanine. A significant fate of tyrosine is conversion into catecholamines, e.g., dopamine, norepinephrine and epinephrine (Zhao et al., 2013). The reduced level of phenylalanine in this study was indicative of the disturbance in phenylalanine metabolism, which is associated with the phenylalanine 4-hydroxylase, an enzyme responsible for converting phenylalanine into tyrosine in kidney and liver (Duranton et al., 2014). Methionine is an indispensable dietary amino acid required for the normal growth and development of humans, other mammals and avian species. Methionine is required for the synthesis of cysteine and forms homocysteine through a demethylation reaction. Homocysteine is a well-established biomarker of renal function, and as its concentration increases in kidney tissues, renal function worsens (Taes et al., 2003). Up-regulation of methionine and down-regulation of phenylalanine observed in this study, suggested the occurrence of renal damage.

In addition, significant decrease of valine and leucine (branched-chain amino acids, BCAAs), as well as phenylalanine and tyrosine (aromatic amino acids, AAAs) were also observed in the UUO rats. BCAAs and AAAs were essential precursors for protein synthesis and energy production (Xu et al., 2015), their level decrease also suggested an increased protein synthesis to repair the damaged membrane protein structure.

### UUO induced oxidative stress

Uric acid is an intermediate metabolite in purine metabolism, while xanthine oxidase (XO) controls the rate limiting step of purine catabolism by converting xanthine to uric acid, along with the generation of reactive oxygen species (ROS; Desco et al., 2002). Moreover, uric acid can be further oxidized to allantoin by various ROS, and allantoin has been considered as a candidate biomarker for oxidative stress related to renal damage(Kand'ár and Žáková, 2008). Thus, high level of allantoin suggested the renal dysfunction probably due to a disorder of purine metabolism and activation of the XO pathway in UUO rats.

Recent studies have shown the major pathways involving the development of renal fibrosis after the unilateral ureteral was ligated. Initially, the interstitium is infiltrated by monocytes and macrophages, which activate inflammatory cytokine (TNF-α) and pro-fibrotic cytokine (TGF-β). Angiotensin II is stimulated by monocytes, which activate the production of NF-κB and ROS, causing further injury to the renal tubule and interstitium (Chevalier et al., 2009). In this study, the significantly increased score of tubular dilatation and the expression of TGF-β, as well as the induced oxidative stress in UUO rats suggested progressive interstitial fibrosis of the obstructive kidney due to tubular cell injury and apoptosis.

In addition, oxidative stress may be associated with free radical production induced by hypoxic conditions because of ligature. HGF is a pleiotropic factor that plays an important role in tubular repair and regeneration after acute renal injury (Liu, 2004). Growing evidence indicates that HGF is a regenerative, cytoprotective molecule as well as an endogenous antifibrotic factor that shows an impressive efficacy in ameliorating tissue fibrosis in a wide variety of animal models, regulating motility, mitogenesis, and morphogenesis in a cell type-dependent fashion (Liu and Yang, 2006). HGF and TGF-β1 regulate diversified cellular functions and often act antagonistically against each other. It has been reported that the reciprocal imbalance between TGF-β1 and HGF is closely involved in the progression of tissue fibrosis. Previous studies also indicated that TGF-β1 and HGF are counteracting in their biological activities (Mizuno et al., 2001). However, the relationship between the significantly changed metabolites and the over-expressed TGF-β1 as well as inhibited-expressed HGF in UUO remains unknown and needs further investigation.

### Disturbed energy metabolism

One of the most important functions of the kidney is the transfer of sodium ions from the tubular fluid to the blood; energy demands for this process are provided by mitochondrial aerobic and anaerobic metabolism (Niemann and Serkova, 2007). 3-HB, one of ketone bodies, is the metabolic products of fatty acids β-oxidation and can be utilized as an alternative energy source when glucose is limited (Lei et al., 2008). Levels of ketone bodies can be increase when acetyl-CoA derived from β-oxidation of free fatty acid exceeds the capacity of the TCA cycle (Liao et al., 2007). Elevation of 3-HB indicates that UUO affected the activities of the key enzymes involved in the metabolism of ketone bodies and induced a shift in energy metabolism toward fatty acid β-oxidation and ketogenesis due to injury to the proximal tubule.

A related study (Hauet et al., 2000) of renal injury in a cold ischemia model demonstrated that elevated urinary level of lactate was indicative of acute tubular necrosis and damage to proximal tubular metabolism due to enhanced anaerobic glycolysis, to replenish insufficient injured renal energy production. In this study, the observed higher level of lactate in the kidney tissue of UUO rats may be the evidence of enhanced anaerobic glycolysis, which implied renal injury. It was also observed in the study of acute liver injury, where the increased level of lactate and the respective enhanced glycolysis confirmed the liver damage (Bernal et al., 2002; Waters et al., 2005).

### Perturbed osmolyte metabolism for RIF

As one of its normal function, the kidney is known to make use of small molecule organic osmolytes in maintaining osmotic balance. The key players in osmotic regulation included taurine, myo-inositol, betaine, and TMAO (Peiqiu et al., 2009). Such organic osmolytes are accumulated in kidney tissue to maintain the structural and functional integrity of cell membranes thus alleviating the perturbations and toxic effects of high salt or other stresses (Bagnasco et al., 1986; Burg, 1997). It is not surprising that the decline of renal function may affect the osmotic regulation. Thus, the decreased TMAO may also contribute to tissue damage result from cellular osmotic imbalances of UUO rat kidneys. The similar result was also observed in polycystic kidney disease (Ogbron et al., 1997).

### Alteration in adenine metabolism

Adenine is derived from the nucleotide inosine monophosphate (IMP), which is synthesized on a pre-existing ribose phosphate through a complex pathway using substrates from the amino acids glycine, glutamine, and aspartic acid, as well as fused with the enzyme tetrahydrofolate (Wang et al., 2002). Adenine is a nitrogen heterocycles present at very low level in blood, and excess adenine is transformed to 2,8-dihydroxyadenine when oxidized by xanthine dehydrogenase in the kidney. The low solubility of 2,8-dihydroxyadenine can lead to its precipitation in the tubules of the kidney. According to Yokzawa et al., excretion of nitrogen compounds is suppressed by renal tubular occlusion due to 2,8-dihydroxyadenine. This, in turn, leads to accumulation of urea nitrogen and creatinine in the blood (Zhao et al., 2013).

Adenine forms adenosine, a nucleoside, when attached to ribose, and it forms adenosine triphosphate (ATP), a nucleotide, when three phosphate groups are added to adenosine. ATP is used in cellular metabolism as one of the basic methods of transferring chemical energy between chemical reactions, maintaining energy balance (Ereciñska and Wilson, 1982). Moreover, adenosine is an important signaling molecule that is induced under ischemic and hypoxic conditions (Fredholm, 2007). Thus, the increased adenosine in the right kidney indicated renal lesions due to ischemia or hypoxia, and decreased level of adenosine in the left kidney may result from increased cell apoptosis, functional destruction and ATP depletion (Tang et al., 2015) in left ligated kidney.

In this study, the reduced level of adenosine in the UUO rats indicated the pathway that adenine forming adenosine was blocked, which further resulted in ATP depletion and energy balance destruction. Thus, adenine became 2,8-dihydroxyadenine when the left ureter was ligated, which was in agreement with the accumulation of urea nitrogen and creatinine in the serum.

### Supposed underlying molecular mechanism of RIF based on comparison of perturbations in multiple biochemical processes between the left and right kidney of UUO rats

Due to the occurrence of moderate RIF, the size of the right kidney adaptively increased as the rats with RIF grow, due to the obstruction of left ureter and renal functional loss. It may be caused by the increased renal blood flow and amino acid delivery to the remaining right kidney, followed by elevated amino acid content and protein synthesis, and activation of a class III PI3K/mTORC1/S6K1 pathway (Liao et al., 2007). From the degree of changed metabolites (α), the molecular mechanism of RIF was characterized by the significant alteration of amino acid metabolism (lysine, tyrosine, leucine, valine, phenylalanine), adenine metabolism (adenine and adenosine), energy metabolism, osmolyte (TMAO), and oxidative stress (allantion), and progressed to end-stage deterioration of renal function even to renal failure.

## Summary

UUO in the rodents is a classic model of RIF that is similar in many aspects to renal failure observed clinically (Chevalier et al., 2009; Boor et al., 2010). Kidney tissue targeted metabolic profiling of UUO rats can elucidate the underlying molecular mechanism of RIF from the perturbations in multiple biochemical processes. In this study, NMR based metabolomic analysis of bilateral kidney tissue, along with biochemical parameters, histopathologic, and immunohistochemistry examination, were employed to explore the molecular mechanism of RIF on UUO rats. The results showed that amino acid metabolism, adenine metabolism, energy metabolism, osmolyte change, and oxidative stress were correlated with the pathogenesis of RIF. For the right kidney of UUO rats, the histology and immunohistochemistry showed slight changes (low tubular injury score and little interstitial collagen deposition, and the similar level of HGF protein expression to that of SO group), however, moderate RIF might also happen in the contralateral bilateral kidney, which was evidenced by altered metabolic profile and disturbed biochemical processes. The results provided further information on the molecular mechanism of RIF, and also demonstrated the sensitivity of metabolomic analysis.

## Author contributions

ZL designed experiments, AL collected and analyzed the raw data. ZL and AL wrote the manuscript. JG and HL analyzed and discussed the data. JG contributed analytic tools. ZL and XQ revised the paper.

### Conflict of interest statement

The authors declare that the research was conducted in the absence of any commercial or financial relationships that could be construed as a potential conflict of interest.

## Supplementary material

Table S1 **Biochemical data between UUO and SO rats**.

Table S2 **Histopathological examination by H&E and Masson**.

Table S3 **^1^H NMR signal assignments of metabolites in kidney tissue extracts of rats**.

Table S4 **Three different methods to identify significant metabolites between A and C groups**.

Figure S1 **Representative photographs of TGF-**β**1 and HGF visualized by immunohistochemical staining in different groups: (A,E) the left kidney of UUO rat, (B,F) the right kidney of UUO rat, (C,G) the left kidney of SO rat and (D,H) the right kidney of SO rat**.

Figure S2 **Representative heteronuclear single quantum coherence (HSQC) spectra**.

Figure S3 **Representative ^1^H-^1^H-correlated spectroscopy (COSY) spectra**.

Figure S4 **(A) PCA score plot showing a separation of A and C groups, (B) Permutation test**.

Figure S5 **OPLS-DA scores plot (A) and coefficient-coded S-plot (B) based on the ^1^H NMR spectra of SO and UUO rats**. The coefficient-coded S-plot corresponding to OPLS-DA revealing the metabolites with large intensities responsible for the discrimination of the corresponding score plot.

Figure S6 **Permutation test generated from the PLS-DA model distinguishing C and D of SO rats**.

Figure S7 **Biplot obtained from the PLS describing the correlation among all variables, including the potential biomarkers of RIF (X variables), the grouping or cluster (observation), and the data of clinical biochemical and histopathology (Y variables)**.

## Abbreviations

^1^H NMR: Proton nuclear magnetic resonance
UUO: unilateral ureteral obstruction
SO: sham-operated
RIF: renal interstitial fibrosis
EMT: epithelial-mesenchymal transition
α-SMA: α-smooth muscle actin
TGF-β1: transforming growth factor-β1
HGF: hepatocyte growth factor
MCP-1: monocyte chemoattractant protein-1
TEMT: tubular epithelial-myofibroblast transition
ROS: reactive oxygen species
BUN: blood urea nitrogen
ALB: albumin
Scr: serum creatinine
H&E-stained: Hematein Eosin-stained
HSQC: Heteronuclear Single Quantum Correlation
COSY: Correlation Spectroscopy
TMAO: Trimethylamine-N-oxide
PCA: Principal component analysis
PLS-DA: partial least squares discriminant analysis
OPLS-DA: orthogonal projection to latent structure with discriminant analysis
VIP: Variable importance in the projection
KEGG: Kyoto Encyclopedia of Genes and Genomes
BCAAs: branched-chain amino acids
XO: xanthine oxidase
TNF-α: Tumor Necrosis Factor-α
NF-κB: nuclear factor kappa-light-chain-enhancer of activated B cells
TCA: tricarboxylic acid
IMP: inosine monophosphate
ATP: adenosine triphosphate.
