# Supplementary material for: Kidney Tissue Targeted Metabolic Profiling of Unilateral Ureteral Obstruction Rats by NMR
Source: Front Pharmacol. 2016 Sep 15;7:307. doi: 10.3389/fphar.2016.00307 (PMC5023943; doi:10.3389/fphar.2016.00307)
Supplement: Table S1 — Biochemical data between UUO and SO rats. [file Table1.DOCX]

**Table S1.** Biochemical data between UUO and SO rats.

|  | SO | UUO |
| --- | --- | --- |
| Scr | 19.40 ± 1.68 | 27.574 ± 3.69*** |
| BUN | 7.97± 1.17 | 11.10 ± 1.42*** |
| ALB | 36.94 ± 1.12 | 35.43 ± 1.58* |

Compared to SO, **p*< 0.05, ***p*< 0.01, ****p*< 0.001
