# Supplementary material for: Kidney Tissue Targeted Metabolic Profiling of Unilateral Ureteral Obstruction Rats by NMR
Source: Front Pharmacol. 2016 Sep 15;7:307. doi: 10.3389/fphar.2016.00307 (PMC5023943; doi:10.3389/fphar.2016.00307)
Supplement: Table S2 — Histopathological examination by H&E and Masson. [file Table2.DOCX]

**Table S2.** Histopathological examination by H&E and Masson.

|  | A | B | C | D |
| --- | --- | --- | --- | --- |
| Tubular injury score | 27.0 | 7.3 | 4.9 | 3.6 |
| Interstitial collagen deposition (%) | 23.8 | 14.6 | 5.3 | 4.3 |
