# Supplementary material for: Kidney Tissue Targeted Metabolic Profiling of Unilateral Ureteral Obstruction Rats by NMR
Source: Front Pharmacol. 2016 Sep 15;7:307. doi: 10.3389/fphar.2016.00307 (PMC5023943; doi:10.3389/fphar.2016.00307)
Supplement: Table S3 — 1H NMR signal assignments of metabolites in kidney tissue extracts of rats. [file Table3.DOCX]

**Table S3.** ^1^H NMR signal assignments of metabolites in kidney tissue extracts of rats.

| Keys | Metabolites | Moieties | δ^1^H (multiplicity) |
| --- | --- | --- | --- |
| 1 | Valine *^b, c, d, e, f^* | *γ*CH_3_, *γ'*CH_3_ | 0.99(d^a^), 1.05(d) |
| 2 | leucine *^b, c, d, e, f^* | *δ*CH_3_, *δ*'CH_3_, *α*CH | 0.96(d), 0.97(d), |
| 3 | isoleucine *^b, c, d, f^* | *δ*CH_3_, *β′*CH_3_, *β*CH | 0.94(t), 1.01(d), 1.99(m) |
| 4 | 3-Hydroxybutyrate *^b, c^* | *γ*CH_3_ | 1.21(d) |
| 5 | lactate *^b, c, e, f^* | *α*CH, *β*CH_3_ | 1.33(d), 4.12(q) |
| 6 | alanine *^b, c, f^* | *β*CH_3_ | 1.49(d) |
| 7 | lysine *^b, c, d, e, f^* | *γ*CH_2_, *β*CH_2_, *δ*CH_2_ | 1.49(m), 1.73(m), 1.92(m) |
| 8 | arginine *^b, c^* | *γ*CH_2_, *β*CH_2_, *δ*CH_2_, *α*CH | 1.68(m), 1.90(m), 3.24(t), 3.76(t) |
| 9 | acetate *^b^* | CH_3_ | 1.93(s) |
| 10 | glutamate *^b, c, d, e, f^* | βCH_2_, *γ*CH_2_ | 2.06(m), 2.12(m), 2.35(m) |
| 11 | methionine *^b, d, e, f^* | *γ*CH_2_, S-CH_3_ | 2.14(s), 2.65(t) |
| 12 | glutamine *^b, c^* | *β*CH_2_, *γ*CH_2_ | 2.13(m), 2.44(m), 3.77(t) |
| 13 | succinate *^b^* | CH_2_ | 2.41(s) |
| 14 | citrate *^b, c^* | CH_2_, CH_2_^'^ | 2.54(d), 2.67(d), |
| 15 | dimethylamine (K) *^b^* | CH_3_ | 2.74(s) |
| 16 | aspartate *^b, f^* | *β*CH_2_, *β*CH_2_^'^ | 2.69(dd), 2.82(dd) |
| 17 | asparagine *^b, d, e, f^* | *β*CH_2_, *β*CH_2_^'^ | 2.88(dd), 2.96(dd) |
| 18 | *scyllo*-Inositol *^b, c^* | CH | 3.37 (s) |
| 19 | creatine *^b, f^* | CH_3_, CH_2_ | 3.04(s), 3.94(s) |
| 20 | creatinine *^b, f^* | CH_3_, CH_2_ | 3.04(s), 4.06(s) |
| 21 | choline *^b, e^* | *β*CH_2_, N-CH_3_ | 3.21(s) |
| 22 | phosphocholine *^b, c^* | N-CH_3_ | 3.22(s) |
| 23 | trimethylamine-N-oxide *^b^* | CH_3_ | 3.27(s) |
| 24 | taurine *^b, c, e, f^* | S-CH_2_, N-CH_2_ | 3.27(t), 3.42(t) |
| 25 | betaine *^b, c, f^* | CH_3_, CH_2_ | 3.27(s), 3.91(s) |
| 26 | myo-Inositol *^b, c, e^* | 1-CH | 3.54(dd), 3.63(t), 4.07(t) |
| 27 | β-glucose *^b, d, e^* | 1-CH, 6-CH′, 5-CH | 4.66(d) |
| 28 | α-glucose *^b, d, e, f^* | 1-CH, 6-CH’ | 5.25(d) |
| 29 | glycine *^b^* | CH_2_ | 3.57(s) |
| 30 | glycerol *^b, c^* | CH(OH) | 3.56(dd), 3.64(dd) |
| 31 | allantoin *^b^* | CH | 5.39(s) |
| 32 | uracil *^b, e, f^* | 5-CH, 6-CH | 5.81(d), 7.55(d) |
| 33 | uridine (M) *^b, c, e, f^* | 5-CH, 1-CH, 6-CH | 5.91(d), 5.92(d), 7.89(d) |
| 34 | cytidine *^b, c, d, e, f^* | 2-CH, 3-CH | 6.07(d), 7.85(d) |
| 35 | fumarate *^b, f^* | CH | 6.53(s) |
| 36 | tyrosine *^b, c, d, e, f^* | 3,5-CH, 2,6-CH | 6.91(d), 7.20(d) |
| 37 | histidine *^b, c^* | 2-CH, 4-CH | 7.11(s), 7.92(s) |
| 38 | phenylalanine *^b, d, f^* | 2,6-CH, 3,5-CH, 4-CH | 7.33(m), 7.38(m), 7.43(m) |
| 39 | nicotinamide *^b, d, e, f^* | 2-CH, 4-CH, 5-CH, 6-CH | 7.60(dd), 8.72(dd), 8.95(d) |
| 40 | xanthine *^b^* | CH | 7.91(s) |
| 41 | adenine *^b, c^* | 2-CH, 8-CH | 8.20(s), 8.22(s) |
| 42 | formate *^b^* | CH | 8.46(s) |
| 43 | tryptophan *^b, c, d, e, f^* |  | 7.19(t), 7.29(t), 7.54(d), 7.74(d) |
| 44 | nicotinamide mononucleotide (NMN) *^b, c^* | 6-CH, 2-CH | 8.31(t), 9.00(d), 9.31(d), 9.64(s) |
| 45 | ethanolamine *^b, c^* | CH_2_NH_2_ | 3.15(t) |
| 46 | adenosine *^b, c^* | 3-C’H(ribose),  1-C’H(ribose), 8-CH(ring), 2-CH(ring) | 4.45(t), 6.10(d), 8.25(s), 8.35(s) |
| 47 | Glycerophosphocholine (GPC) *^b, c^* | N-CH_3_, βCH_2_ | 3.23(s), 3.68(t) |
| 48 | guanidoacetic acid *^b, c^* | N-CH_2_ | 3.78(s) |
| 49 | proline *^b, c^* | *γ*CH_2_, *β*CH_2_, *δ*CH_2_, *α*CH | 1.99(m), 2.07(m); 2.34(m); 3.32(m), 3.41(m); 4.13(dd) |
| 50 | malic acid *^c,^* *^f^* | 1-CH, 2-CH_2_ | 2.39(dd), 2.70(dd), 4.32(d) |
| 51 | glycolate *^f^* | CH_2_ | 3.96(s) |
| 52 | serine *^c, f^* | CH, CH_2_ | 3.87(dd, dd), 3.99(dd) |
| 53 | tartaric acid *^f^* | CH | 4.32(s) |
| 54 | aminoisobutyric acid *^f^* | CH_3_ | 1.50(s) |
| 55 | creatine phosphate *^f^* | CH_3_, CH_2_ | 3.05(s), 3.93(s) |
| 56 | L-carnitine *^c, f^* | *α*CH_2_, N-CH_3_, *γ*CH_2_, *β*CH | 2.45(dd), 3.25(s), 3.43(dd), 4.57(dd, dd) |
| 57 | γ-aminobutyric acid *^c, f^* | *β*CH_2_, *α*CH_2_, *γ*CH_2_ | 1.91(m), 2.31(t), 3.04(t) |
| 58 | 4-guanidinobutyric acid *^f^* | *β*CH_2_, *α*CH_2_, *γ*CH_2_ | 1.78(m), 2.25(t), 3.16(t) |

*^a^*Multiplicity for ^1^H resonances: s, singlet, d, doublet, t, triplet, m, multiplet, dd, doublet of doublet.

Metabolites annotated as to how they are identified, *^b^*refer to literature; *^c^*according to database; *^d^*identified by manual HSQC; *^e^*identified by COSY; *^f^*identified by COLMAR^13^C-^1^H HSQC Query.
