# Supplementary material for: Kidney Tissue Targeted Metabolic Profiling of Unilateral Ureteral Obstruction Rats by NMR
Source: Front Pharmacol. 2016 Sep 15;7:307. doi: 10.3389/fphar.2016.00307 (PMC5023943; doi:10.3389/fphar.2016.00307)
Supplement: Table S4 — Three different methods to identify significant metabolites between A and C groups. [file Table4.DOCX]

**Table S4.** Three different methods to identify significant metabolites between A and C groups.

| No. | Compound | VIP | coefficient | t-test |
| --- | --- | --- | --- | --- |
| 1 | methionine | 1.1092 | 90.554 | 5.26E-07 |
| 2 | aspartate | 1.1707 | 98.843 | 8.65E-09 |
| 3 | uracil | 1.0282 | 79.625 | 1.58E-05 |
| 4 | 3-HB | 1.0581 | 83.65 | 5.33E-06 |
| 5 | TMAO | 1.0915 | 88.166 | 1.27E-06 |
| 6 | phenylalanine | 1.1267 | 92.914 | 7.65E-07 |
| 7 | lactate | 1.0819 | 86.862 | 1.97E-06 |
| 8 | allantoin | 1.1343 | 93.936 | 1.25E-07 |
| 9 | leucine | 1.1238 | 92.515 | 2.36E-07 |
| 10 | valine | 1.1793 | 100 | 3.98E-09 |
| 11 | lysine | 1.1053 | 90.015 | 6.47E-07 |
| 12 | adenosine | 1.1476 | 95.732 | 5.18E-08 |
| 13 | hypoxanthine | 1.0158 | 77.946 | 2.39E-05 |
| 14 | tyrosine | 1.0987 | 89.126 | 9.01E-07 |
