# Supplementary figures and images for: Kidney Tissue Targeted Metabolic Profiling of Unilateral Ureteral Obstruction Rats by NMR
Source: Front Pharmacol. 2016 Sep 15;7:307. doi: 10.3389/fphar.2016.00307 (PMC5023943; doi:10.3389/fphar.2016.00307)

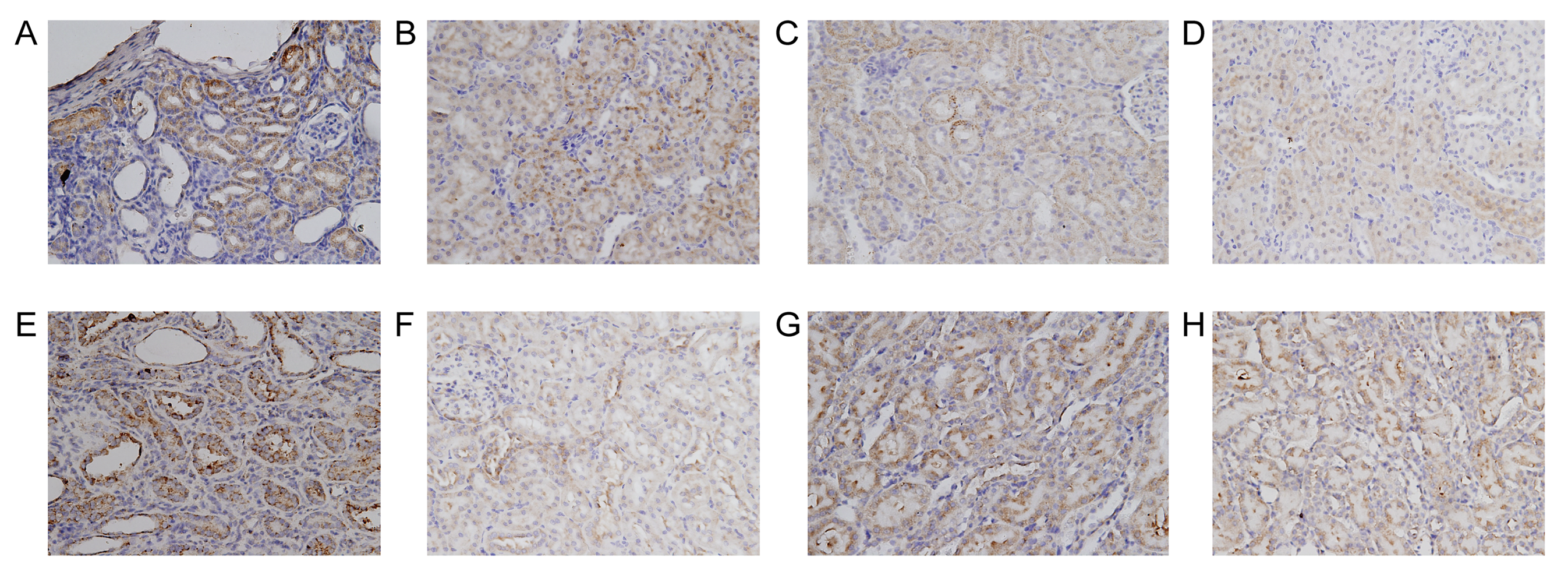

Supplement: Figure S1 — Representative photographs of TGF-β1 and HGF visualized by immunohistochemical staining in different groups: (A,E) the left kidney of UUO rat, (B,F) the right kidney of UUO rat, (C,G) the left kidney of SO rat and (D,H) the right kidney of SO rat. [file Image1.TIF]

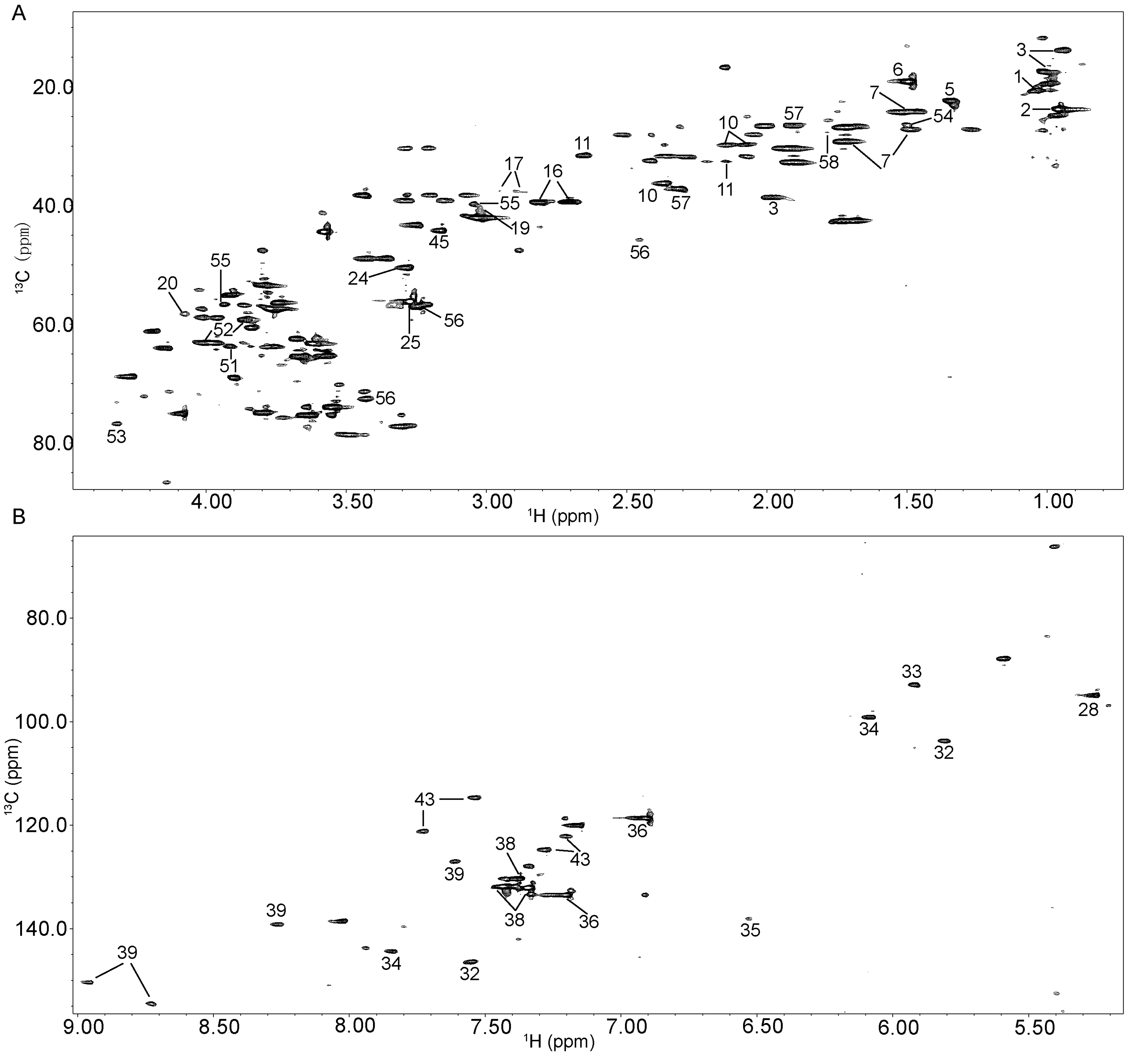

Supplement: Figure S2 — Representative heteronuclear single quantum coherence (HSQC) spectra. [file Image2.TIF]

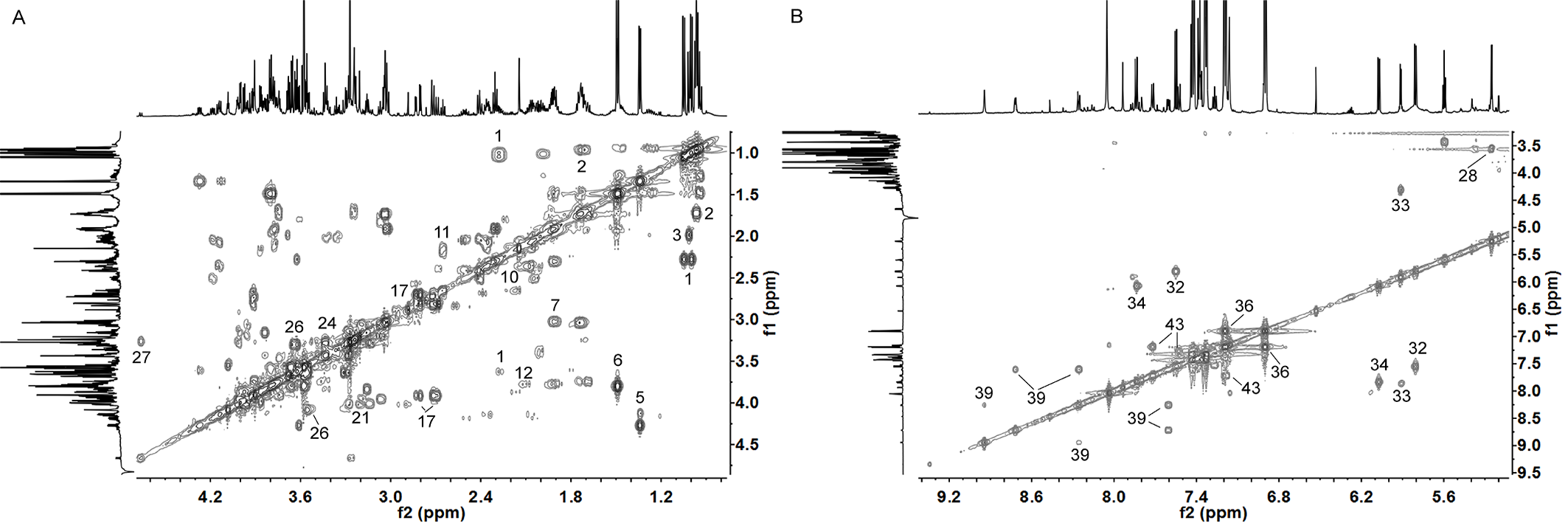

Supplement: Figure S3 — Representative 1H-1H-correlated spectroscopy (COSY) spectra. [file Image3.TIF]

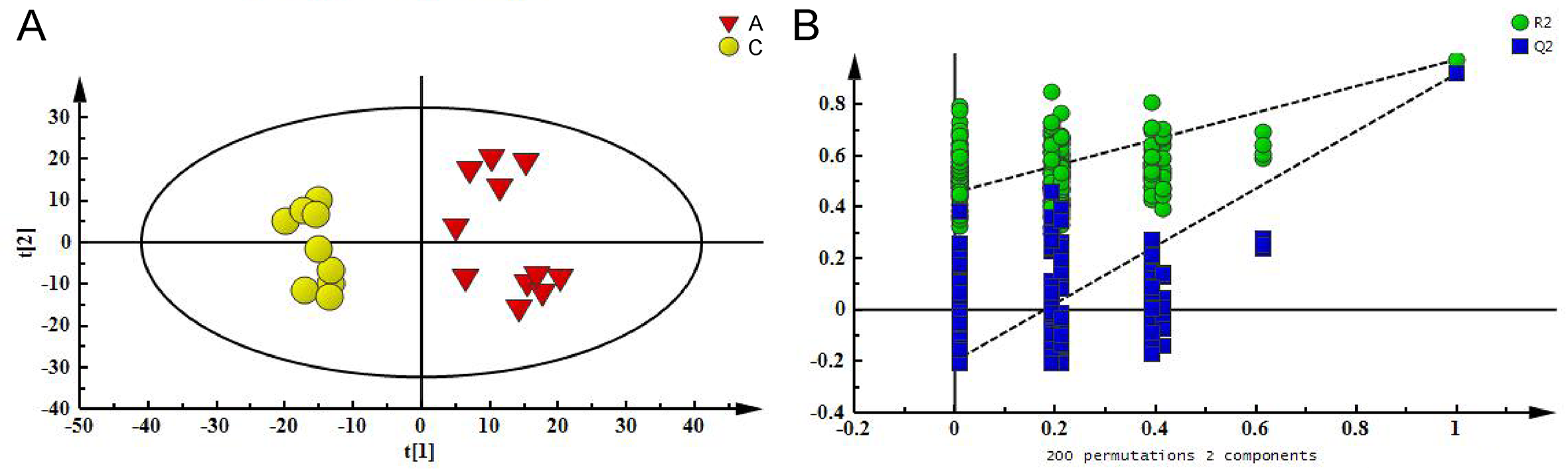

Supplement: Figure S4 — (A) PCA score plot showing a separation of A and C groups, (B) Permutation test. [file Image4.TIF]

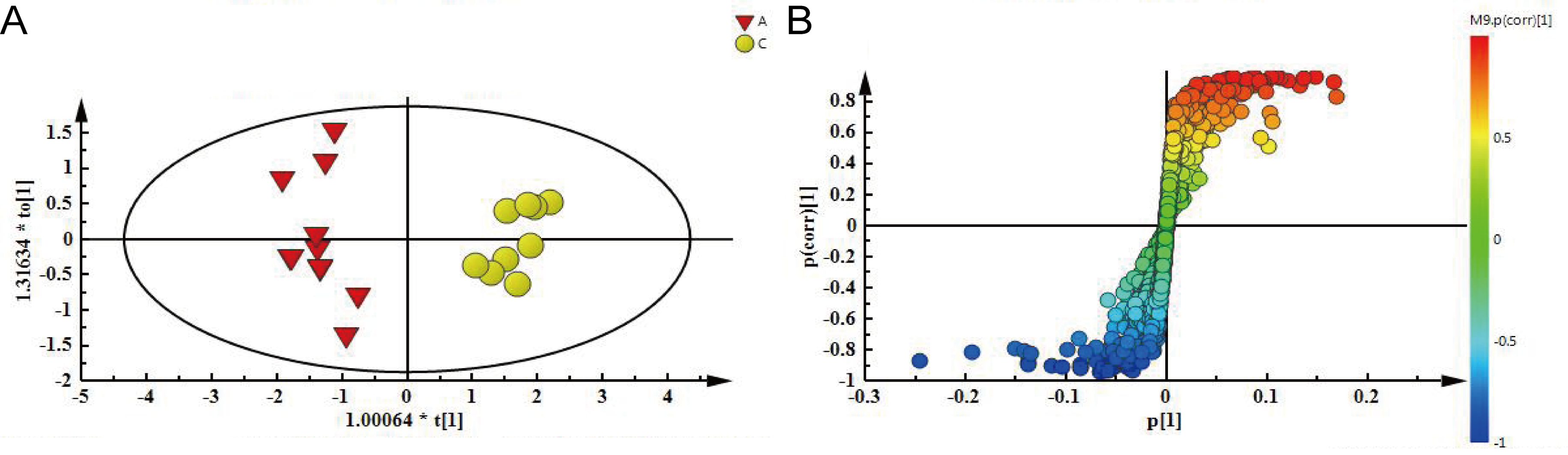

Supplement: Figure S5 — OPLS-DA scores plot (A) and coefficient-coded S-plot (B) based on the 1H NMR spectra of SO and UUO rats. The coefficient-coded S-plot corresponding to OPLS-DA revealing the metabolites with large intensities responsible for the discrimination of the corresponding score plot. [file Image5.TIF]

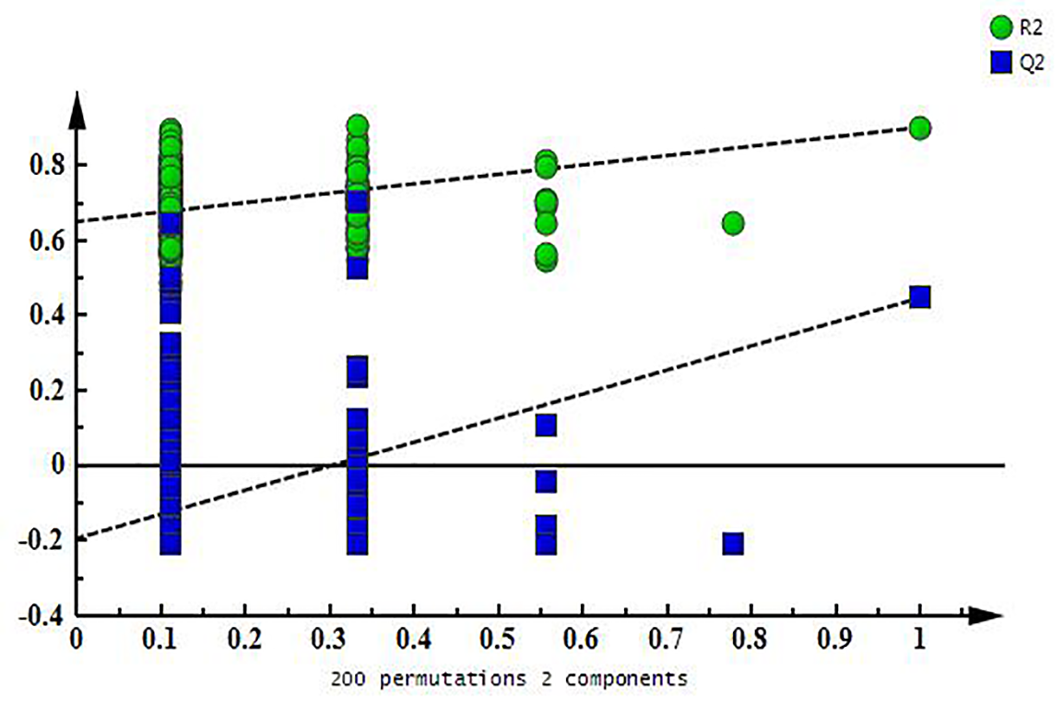

Supplement: Figure S6 — Permutation test generated from the PLS-DA model distinguishing C and D of SO rats. [file Image6.TIF]

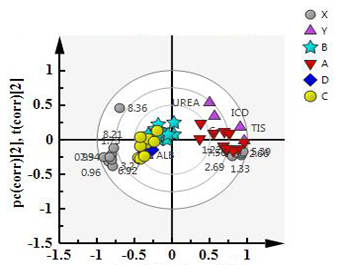

Supplement: Figure S7 — Biplot obtained from the PLS describing the correlation among all variables, including the potential biomarkers of RIF (X variables), the grouping or cluster (observation), and the data of clinical biochemical and histopathology (Y variables). [file Image7.TIF]
